# A Study on the Influence of the Nozzle Lead Angle on the Performance of Liquid Metal Electromagnetic Micro-Jetting

**DOI:** 10.3390/mi7120220

**Published:** 2016-12-05

**Authors:** Zhiwei Luo, Gaofeng Zheng, Lingyun Wang

**Affiliations:** 1 Department of Mechanical and Electrical Engineering, Xiamen University, Xiamen 361005, China; 2 School of Mechanical and Automotive Engineering, Xiamen University of Technology, Xiamen 361024, China; 3 Department of Instrumental and Electrical Engineering, Xiamen University, Xiamen 361005, China; zheng_gf@xmu.edu.cn

**Keywords:** electromagnetic micro-jetting, micro-nozzle, lead angle, liquid metal, Lorentz force

## Abstract

To improve the jetting performance of liquid metals, an electromagnetic micro-jetting (EMJ) valve that realizes drop-on-demand (DOD) jetting while not involving any valve core or moving parts was designed. The influence of the lead angle of the nozzle on the jetting of liquid metal gallium (Ga) was investigated. It was found that the Lorentz force component parallel to the nozzle that jets the electrified liquid Ga is always larger than its internal friction; thus, jet can be generated with any lead angle but with different kinetic energies. Experimental results show that the mass of the jetting liquid, the jetting distance, the initial velocity of the jet, and the resulting kinetic energy of the jet increase first and then decrease. When the lead angle is 90°, the mass of the jetting liquid and the kinetic energy are at their maximum. When the angle is 80°, the initial velocity achieves its maximum, with a calculated value of 0.042 m/s. Moreover, very close and comparatively high kinetic energies are obtained at 80° and 90°, indicating that angles in between this range can produce a preferable performance. This work provides an important theoretical basis for the design of the EMJ valve, and may promote the development and application of micro electromagnetic jetting technology.

## 1. Introduction

Liquid metal droplet jetting technology was not developed until the 1990s [1]. This technology is highly automatic, environmentally friendly, low-cost, and non-contact [2,3] and can precisely deposit metallic materials such as copper [4], aluminum alloy [5], and tin lead alloy [6] with high efficiency. Its application can be found in many fields, including but not limited to complex metal forming, micro electro mechanical system (MEMS), 3D packaging [7,8,9,10], flexible electronics, and microcircuit fast printing [11].

To date, various micro-jetting techniques have been developed for different kinds of metal materials. Driving modes include the pneumatic driving mode, the stress wave driving mode, the electromagnetic force driving mode, and the laser melting mode [12]. According to the jet behavior, they can be classified into a drop-on-demand (DOD) jet and a continuous-ink jet (CIJ). Yuan et al. [13] have developed a pneumatic drive jetting apparatus to print tin lead alloy and aluminum alloy. Later, they integrated ultrasonic supply powder technology into the apparatus to achieve the molding of heterogeneous metal parts [14]. The melted aluminum alloy was jetted in DOD mode by using the compressed gas as the driving source by Cheng et al. [15] and Cao et al. [16] In addition, Chun et al. [17] and Orme et al. [18] employed the jet crushing technique to break liquid metals into uniform droplet beams and finally obtained a homogeneous spherical metal powder. However, the above techniques are problematic in terms of the size of the liquid droplet and/or the jetting frequency. The minimum diameter of the liquid droplet for the pneumatic driving mode is normally up to 85 μm, with a low stable jetting frequency of only 5 Hz. Using the stress wave driving mode, the diameter can be reduced to 63.8 μm, albeit at a low frequency of 5 Hz. On the other hand, when using the laser melting process, the positioning and the power of the laser needs to be controlled precisely to avoid satellite droplets and to increase the frequency. From the viewpoint of industrial application of liquid metal micro-jetting, the jetting frequency and the stability of the system must be enhanced to print complex patterns, since these two aspects directly affect the efficiency of electronic packaging and 3D printing of metallic materials. For example, some engineering fields require a frequency of more than 150 Hz for specific applications, which is hardly achievable with existing jetting techniques.

To obtain a more satisfactory jetting process, an electromagnetic micro-jetting (EMJ) technique has been proposed. This technique utilizes the Lorentz force field to drive liquid metals to be jetted, where free electrons inside the electrified liquid metal are subjected to a complex field consisting of the electric field and the magnetic field perpendicular to it, and liquid metals are continuously ejected in a specific direction. This technique is characterized by free of moving parts, noise or vibration, meanwhile achieving high jetting frequency. To further demonstrate its performance, EMJ valves with different nozzle lead angles were designed, and experiments were performed.

## 2. Experiment

**Figure 1:**
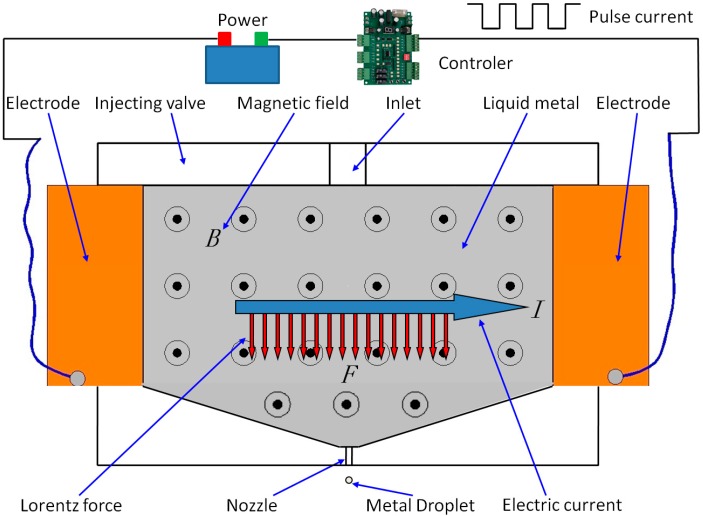
Schematic illustration of electromagnetic micro-jetting (EMJ).

In order to achieve a DOD jetting process, a pulse current is generated from a direct power source via a controller and applied on the liquid metal, and a Lorentz force is instantaneously induced, by which the liquid metal is ejected from the nozzle to form micro droplets, as shown in Figure 1. In this case, the duration (the sum of the rising and falling edge time of pulse current) of the instantaneous Lorentz force on the liquid can be minimized to the microsecond (μs) scale, which can break through the bottleneck of the low jetting frequency currently encountered by other techniques. Moreover, EMJ allows further structural simplification so that the stability of the system can be improved. Experimentally, the following parameters are used: a magnetic field intensity of 0.4 T, a current of approximately 30 A, a voltage of 12 V, a pulse width of 0.5 ms, a distance between electrodes of 36 mm, and an outlet diameter of about 1 mm. According to its working principle, theoretically, EMJ can be used for micro-jetting any kind of liquid metal; however, a high temperature weakens magnetic fields, and more particularly, permanent magnets have their Curie-temperatures. Therefore, the temperature of the liquid metal should not be too high, or a cooling device should be provided to prevent the magnet from overheating. Currently, preferable EMJ metallic materials include gallium, mercury, and a soldering tin alloy. Metallic gallium (Ga) was selected for the experiment because of its low melting point (~29.8 °C) and good stability in the air. With this low melting point, 3D printing of gallium can be realized by micro-jetting, which is a direct 3D printing of metallic parts.

**Figure 2:**
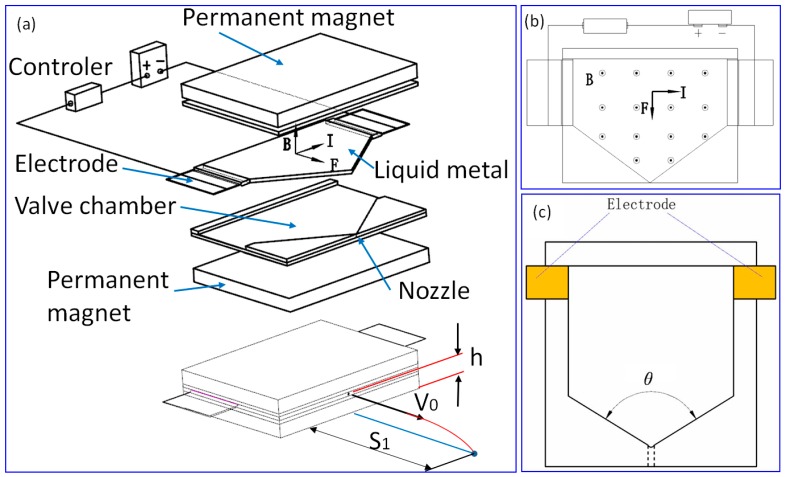
(**a**) 3D view and parameters of the experiment device. (**b**) 2D view of the experiment device. (**c**) Schematics of the jetting valve.

**Figure 3:**
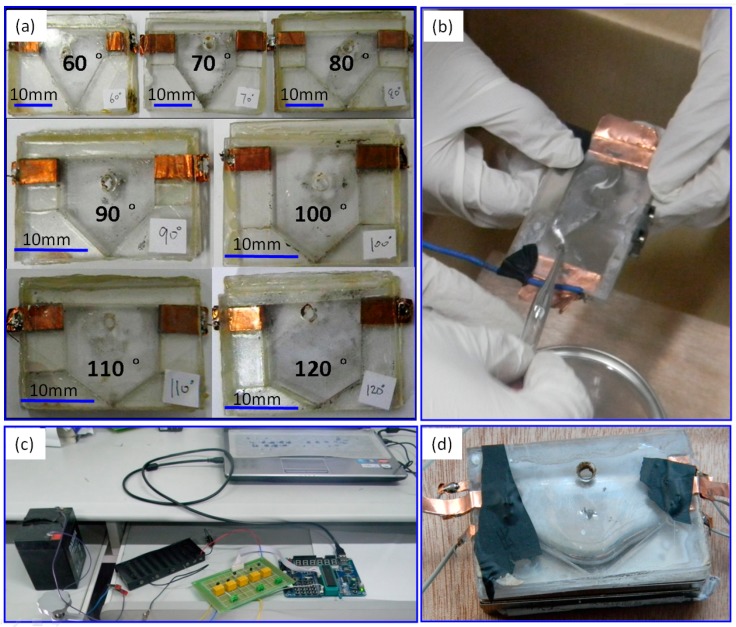
(**a**) Fabricated prototypes of jetting valves with different lead angles (θ). (**b**) Filling gallium into the valve with a dropper. (**c**) The controller and the power source. (**d**) The jetting valve loaded with gallium.

A 3D view and parameters of the experimental device is shown in Figure 2a, a 2D view shown in Figure 2b, and schematics of the jetting valve shown in Figure 2c. The jetting performance of liquid Ga was evaluated by varying the lead angle of the nozzle from 60° to 120°, as shown in Figure 3a. It should be noted that the data are the average of 10 samplings of each experiment, and the jet is considered to follow the horizontal projectile motion at the very beginning. According to Newton’s second law and kinetic energy theorem, Equations (1) and (2) can be deduced, based on which key parameters including the initial velocity (*V*_0_) and the kinetic energy of the droplet (*E*_k_) can be calculated, whereas the jetting distance (*S*_1_) and the mass (*m*) of the jet droplets can be measured in the experiment.

(1) $$V_{0}=S_{1}\times \sqrt{\frac{g}{2h}}$$

(2) $$E_{k}=\frac{1}{2}mV_{0}^{2}=\frac{mgS_{1}^{2}}{4h}$$

where *g* is gravity acceleration; *h* is the nozzle height (*h* = 23.75 mm).

The droplet jetting motion, here, can be taken as the parabolic motion with initial velocity *V*_0_ in the horizontal direction; then, the jetting motion can be derived from Equations (1) and (2).

In order to observe the liquid motion inside the jetting valve, while ensuring current flows through the liquid metal rather through the valve body, clear acrylic boards are used to fabricate the jetting valve. The electrode is fabricated with copper foil so that the current can flow from outside the valve body to the metal liquid inside. All components of the jetting valve are bonded together as a whole with super glue to ensure sealing tightness, as shown in Figure 3a. In the design of this jetting valve, the inlet is located on top of the wide panel, through which gallium is filled in with a dropper, as shown in Figure 3b. The current controller uses an Microcontroller Unit MCU-based (SST89E564RD) control circuit to generate a pulse current with a modulated pulse width, frequency, duty ratio, etc.; the control circuit is isolated from the working current with a relay (HK4100F-DC5V-SHG, HuiKe relay Co., Ltd, Shenzhen, China). A battery UPS 12V6AH (Panasonic Storage Battery(Shenyang) Co., Ltd, Shenyang, China) is used as the power source as shown in Figure 3c. The current of the working circuit is above 30 A due to small loads in the loop. The jetting valve loaded with gallium is shown in Figure 3d. Neodymium magnets are used to provide the valve with a stable magnetic field, with an intensity up to 0.4 T.

## 3. Results and Discussion

**Figure 4:**
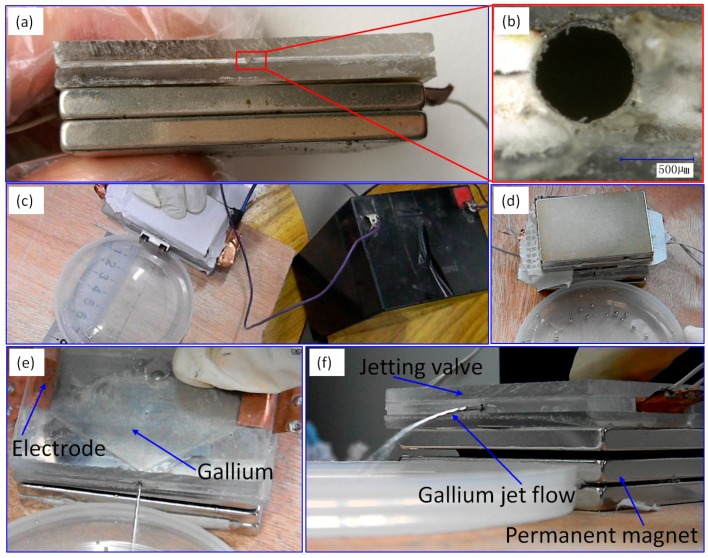
(**a**) The jetting valve. (**b**) The nozzle. (**c**) The experiment platform. (**d**) Droplets collection. (**e**) A top view of the jetting process. (**f**) A side view of the EMJ process.

The jetting valve in working status is shown in Figure 4a, and an enlarged view of the round-shaped nozzle is shown in Figure 4b, with a diameter of 686 μm. In the experiment, the jetting valve is positioned horizontally, and a round plastic disc is placed in front of it as the collector. The jetting distance *S*_1_ is measured with a steel ruler, as shown in Figure 4c. Figure 4d shows metal droplets collected in the disc. A top view of the EMJ process is shown in Figure 4e, and a side view shown in Figure 4f. During the experiment, sometimes the magnet on top was removed to observe the motion of the metal liquid inside the jetting valve, in which circumstance the valve was still functioning. However, all experimental data were collected when both magnets on the top and bottom were installed. The weight of the droplets was measured with an analytical balance. The plastic disc was used as the collecting panel at the beginning, and was then replaced with sticky tapes to prevent droplets from moving around. The experiment was carried out in summer, with a room temperature of 30 °C and a humidity of 50%.

**Figure 5:**
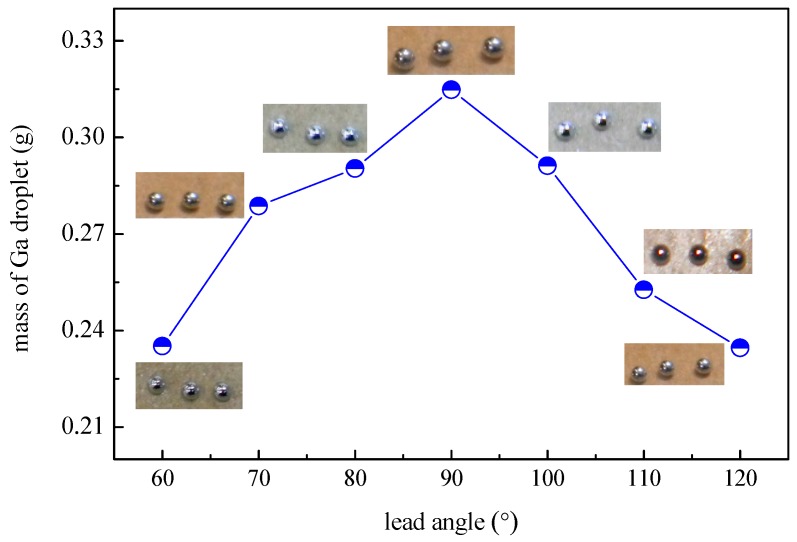
Mass of liquid Ga droplets as a function of the lead angle.

As the lead angle of the nozzle changes, the mass of liquid Ga in the cavity (between the nozzle and electrodes) changes accordingly, so does the Lorentz force component parallel to the nozzle. Figure 5 shows the mass of Ga droplets deposited under different lead angles. It is clear that the mass of droplets increases with the increase in the lead angle from 60° to 90°, but decreases when further increasing the angle (90° < θ ≤ 120°). This trend results from the competition between the Lorentz force component and the internal resistance of liquid Ga in the cavity. The resistance of the liquid Ga increases as the angle increases from 60° to 120°, while the Lorentz force component (parallel to the nozzle) only increases along with the angle from 60° to 90°, and then decreases from 90° to 120°. From 60° to 90°, the Lorentz force component (parallel to the nozzle) exhibits a much larger increase than the resistance of the liquid Ga. Therefore, a greater volume of the droplet is ejected with larger angles, and EMJ at the angle of 90° produces the largest and heaviest droplets (see inset on top).

**Figure 6:**
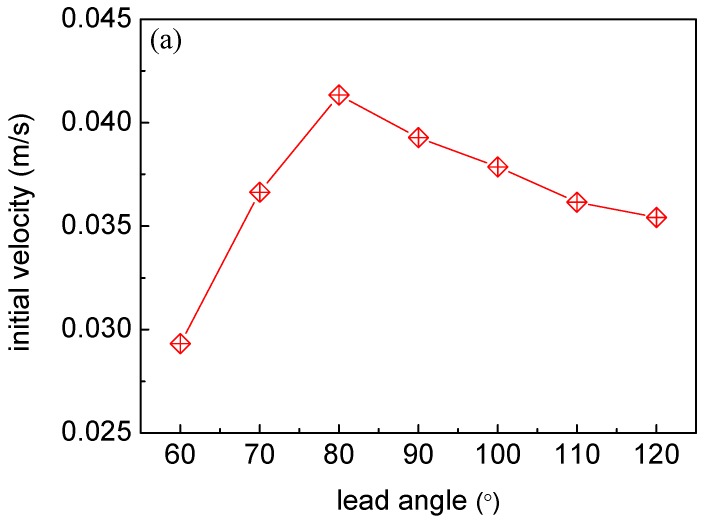

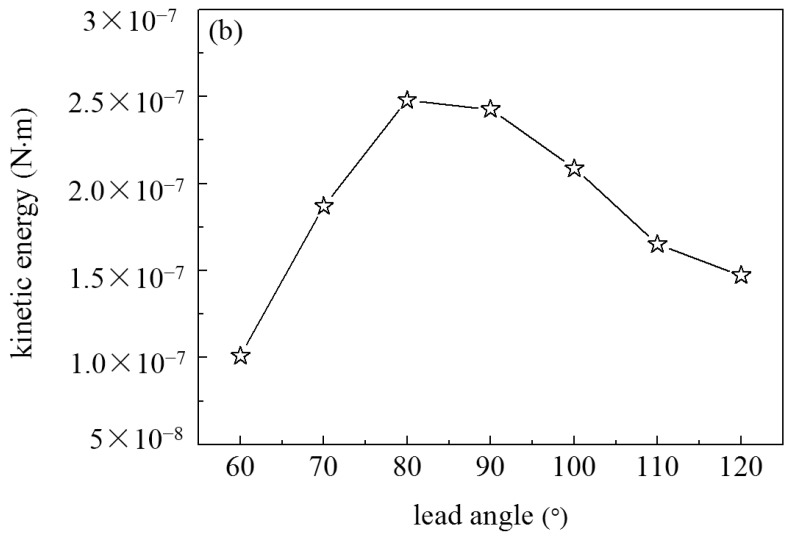
Initial velocity of the jet (**a**) and kinetic energy of the droplet (**b**) as a function of the lead angle respectively.

It is well known that the jetting distance (*S*_1_) is closely correlated with the initial velocity of the jet in a horizontal projectile motion. As mentioned above, *S*_1_ can be directly measured, which shows an increase with the angle growing from 60° to 80°, and then decreases afterwards (figure not shown here). According to Equation (1), the initial velocity (*V*_0_) should have a very similar profile to *S*_1_, as plotted in Figure 6a, where a maximum *V*_0_ of 0.042 m/s is calculated for 80°. Although the Lorentz force component (parallel to the nozzle) achieves the maximum at 90°, the mass of droplets (*m*) also reaches its maximum, resulting in the increased internal friction of the liquid, which could be the reason for a lower *V*_0_ at 90° than at 80°. According to Equation (2), the kinetic energy of the droplet (*E*_k_) can be further determined, as plotted in Figure 6b. Since *E*_k_ is proportional to m (the mass of droplet) and the square of *V*_0_ (initial velocity of the jet), the profile of *E*_k_ is more affected by *V*_0_. Hence, very close and comparatively high values of *E*_k_ are obtained at 80° and 90°, suggesting that these two angles, and that in between, can provide a preferable performance. In addition, the size of the nozzle has a great influence on the diameter of the collected droplets. In the initial design, the nozzle diameter was relatively large (600 μm), which generates droplets with diameters up to 3 mm. Later, a stainless steel nozzle that produces droplets with a diameter of about 350 μm was fabricated (with a diameter of 250 μm).

From the above results, it is reasonable to state that the lead angle has a great influence on jetting performance and the resultant droplets. The production of regular liquid Ga droplets on a micro-scale size preliminarily demonstrates the feasibility of using the EMJ technique to deposit liquid metal in a DOD manner, which we believe has great potential in various manufacturing industries.

## 4. Conclusions

Based on the Lorentz force induced by charges moving in a magnetic field, an EMJ valve capable of drop-on-demand (DOD) jetting is here proposed, and the influence of the nozzle lead angle on the jetting of gallium (Ga) was investigated. Results show that jetting processes, with different initial velocities, occur under all lead angles, because the component of the Lorentz force parallel to the nozzle for the electrified liquid Ga is always greater than its internal friction. The mass of the jet liquid, the jetting distance, the initial velocity of the jet, and the resulting kinetic energy of the jet are found to increase first and then decrease. When the lead angle is 90°, the mass and the kinetic energy are at their maximum, while the initial velocity achieves maximum at 80°. Very close and comparatively high kinetic energies are obtained at 80° and 90°, indicating that a preferable performance can be achieved with angles in between this range. Future improvement on its design allows for better jetting performance of the EMJ technique, which we believe has great potentials in flexible printed circuits, large-scale integrated circuit board welding, and so on.
